# Applied Climate-Change Analysis: The Climate Wizard Tool

**DOI:** 10.1371/journal.pone.0008320

**Published:** 2009-12-15

**Authors:** Evan H. Girvetz, Chris Zganjar, George T. Raber, Edwin P. Maurer, Peter Kareiva, Joshua J. Lawler

**Affiliations:** 1 School of Forest Resources, University of Washington, Seattle, Washington, United States of America; 2 Global Climate Change Program, The Nature Conservancy, Arlington, Virginia, United States of America; 3 Department of Geography and Geology, University of Southern Mississippi, Hattiesburg, Mississippi, United States of America; 4 Department of Civil Engineering, Santa Clara University, Santa Clara, California, United States of America; 5 Worldwide Office, The Nature Conservancy, Seattle, Washington, United States of America; Institut Mediterrani d'Estudis Avançats (CSIC/UIB), Spain

## Abstract

**Background:**

Although the message of “global climate change” is catalyzing international action, it is local and regional changes that directly affect people and ecosystems and are of immediate concern to scientists, managers, and policy makers. A major barrier preventing informed climate-change adaptation planning is the difficulty accessing, analyzing, and interpreting climate-change information. To address this problem, we developed a powerful, yet easy to use, web-based tool called Climate Wizard (http://ClimateWizard.org) that provides non-climate specialists with simple analyses and innovative graphical depictions for conveying how climate has and is projected to change within specific geographic areas throughout the world.

**Methodology/Principal Findings:**

To demonstrate the Climate Wizard, we explored historic trends and future departures (anomalies) in temperature and precipitation globally, and within specific latitudinal zones and countries. We found the greatest temperature increases during 1951–2002 occurred in northern hemisphere countries (especially during January–April), but the latitude of greatest temperature change varied throughout the year, sinusoidally ranging from approximately 50°N during February-March to 10°N during August-September. Precipitation decreases occurred most commonly in countries between 0–20°N, and increases mostly occurred outside of this latitudinal region. Similarly, a quantile ensemble analysis based on projections from 16 General Circulation Models (GCMs) for 2070–2099 identified the median projected change within countries, which showed both latitudinal and regional patterns in projected temperature and precipitation change.

**Conclusions/Significance:**

The results of these analyses are consistent with those reported by the Intergovernmental Panel on Climate Change, but at the same time, they provide examples of how Climate Wizard can be used to explore regionally- and temporally-specific analyses of climate change. Moreover, Climate Wizard is not a static product, but rather a data analysis framework designed to be used for climate change impact and adaption planning, which can be expanded to include other information, such as downscaled future projections of hydrology, soil moisture, wildfire, vegetation, marine conditions, disease, and agricultural productivity.

## Introduction

Climate-change impacts to ecosystems have been well documented [1]. Although there is growing evidence of climate change, natural resource managers have found it difficult to develop management and planning responses to climate change. One reason for this slow response is that there are relatively few tools for translating cutting-edge climate science and climate-model simulations into a form that a manager can work with at a local or regional scale [2]. Although large amounts of data exist regarding how climate has and is projected to change, these data are stored in databases that can be difficult to access. Furthermore, although analytical techniques are available for quantifying the potential effects of these changes, many require significant computing resources and analytical expertise. Scientists, managers, and policy makers (i.e. practitioners) need the ability to assess the potential effects of climate change on specific ecological systems within specific geographic areas at relevant spatial scales.

Here we provide an example of how computer-based technologies can be used to develop tools that make climate-change analysis more accessible, practical, and useful. These technologies include geographic information systems (GIS), statistical analysis platforms (e.g. the R Project), and web-based mapping services (e.g., ArcGIS Online, KML/GML, and SOAP). Specifically, we provide a framework for practical climate-change analysis, and present an internet-based climate data analysis and mapping tool, which we call Climate Wizard.

### Climate Wizard: An Easy-to-Use Tool for Practical Climate Change Analysis

The Climate Wizard is freely available as an interactive website that produces climate-change maps, graphs, and tables (http://ClimateWizard.org). This tool was designed with a range of users in mind. For less technical users, it provides access to a wide range of pre-calculated climate-change analyses based on existing data sets (both past observed and future modeled) that can be explored in a web mapping interface. For more technical users, it can run customized statistical analyses that address relevant ecological questions for specific time periods and within user-specified geographic areas (http://ClimateWizard.org/custom).

**Figure 1:**
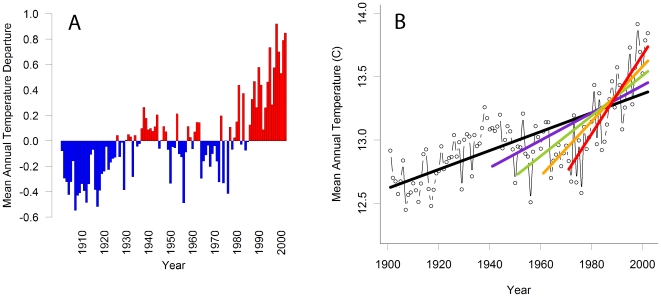
Global temperature change during 1901–2002. (a) Graph of global mean temperature departures relative to the mean during 1901–2002. (b) Graph of trend analysis. The trend analysis over the entire time period (black line) increased at a rate of 0.075°C per decade (0.75°C per century), the trend during 1941–2002 (purple line) increases at a rate of 0.11°C per decade, the trend during 1951–2002 (green line) increases at a rate of 0. 16°C per decade, the trend during 1961–2002 (orange line) increases at a rate of 0.22°C per decade, and the trend during 1971–2002 (red line) increases at a rate of 0.31°C per decade. Data for both graphs were calculated from the CRU TS 2.1 data set [10].

Different scientific, management, and policy questions can require different types of climate data and analyses. The Climate Wizard uses two common approaches for representing climate-change data: (1) comparing climate in a given year or time period to a baseline period (climatic departures or anomalies); and (2) calculating statistical climatic trends over a time period of interest using linear trend analysis (restricted maximum likelihood) that accounts for the time-series nature of climate data (serial temporal autocorrelation). Climatic departures are useful for identifying specific years above or below a threshold value. Such a threshold could represent the climate of a historical time period (e.g., the average of the past century, Figure 1a), or the climatic conditions required by a specific ecological process. For example, the onset of breeding activity of the common toad (*Bufo bufo*) in the United Kingdom is linked to the number of days with mean temperatures above 6°C during the 40 days preceding arrival at the breeding pond [3]. Comparisons to a historical baseline have the advantage of being easy for general audiences to understand. Trend analyses, on the other hand, are useful for assessing continual and incremental change over time and the statistical significance of the trend (Figure 1b).

The Climate Wizard can analyze both past observed and future projected climate data. While analyzing observed (historical) climate data is fairly straight forward, future climate projections from general circulation models (GCMs) can be more complex to analyze. First, there is not one GCM projection of future climate, but rather many projections produced by different GCMs run under a range of greenhouse gas emissions scenarios [4]. Ensemble analyses are frequently used to combine the simulations of multiple GCMs and quantify the range of possibilities for future climates under different emissions scenarios, and the use of an ensemble median (or mean) is an effective means to improve the outcome of climate simulations that is often better than any individual future climate projection [5]. There are many approaches for doing ensemble analyses ranging from simple averaging of different projections to more complex and computationally intensive probability estimation approaches [6]. Second, GCMs often simulate climate at relatively coarse spatial resolutions (e.g., 2.5–3.5 degree grid cells). This spatial resolution is too coarse for addressing many ecological questions. However, Climate Wizard can use high resolution climate data sets created using downscaling techniques that use information from finer resolution data sets of past observed climate to inform how climate will change at finer spatial scales (e.g., [7]; see Methods for more details).

To conduct these analyses, the Climate Wizard requires (1) delineated geographic area(s) over which analyses are to take place; (2) a specified time period over which to calculate a trend analysis or two time periods to compare for a departure analysis; (3) a specified temporal resolution(s), or time domain(s), over which data are to be summarized (annual, seasonal, or monthly); and (4) a list of climate variables of interest (e.g., precipitation, temperature). The Climate Wizard uses ArcGIS [8] SOAP web-services and the R statistical package [9] to access a time-series database of climate information stored on a remote computer server, and then uses the server's computing power to create outputs in the form of graphs, maps, tables, and GIS data layers tailored to the specific climate-change question being asked by the user (see Methods section for more details). Because this tool stores and analyzes the climate data sets on remote computer servers, users of the tool do not need to have fast computers or expensive software to analyze the data, but simply need access to the internet. The outputs can be viewed on the web or downloaded and directly used in presentations, publications, and scientific research. All of the climate maps in this article and in the Supplementary material were created directly from using the Climate Wizard (except Figure 14, and Figure S5 which are analytic derivations of Climate Wizard products).

Here, we use the Climate Wizard toolbox to analyze historic climate data and future climate projections to identify where and when climate has and is projected to change with the greatest magnitude and statistical confidence. We demonstrate how the Climate Wizard can be used to summarize climate-change statistics both historically and for future climate projections, run across months, seasons, and annually, for the entire globe, as well as within latitudinal zones, and individual countries. The ease of use offered by the Climate Wizard makes it possible for users to carry-out analyses that may not be appropriate for certain data sets, or for particular regions or time periods. For this reason, we provide a discussion of some of the assumptions and limitations of the data sets and analysis techniques used here (see Discussion section “Use and misuse of climate data and analyses”). Finally, we discuss how the Climate Wizard is not a static product, but rather a framework that can be extended to address climate-change related questions in a geographic context for a wide range of social and environmental issues.

## Results

### Overview

**Figure 2:**
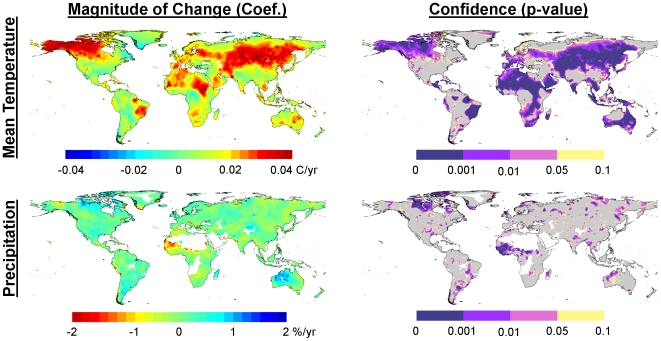
Temperature and precipitation change during 1951–2002. Maps of the magnitude (left) and confidence (right) for annual mean temperature (top) and precipitation trends during 1951–2002 for each 0.5 degree grid cell in the CRU TS 2.1 data set [10]. For temperature trends, warmer colors (yellow-green to red) represent increasing temperatures over time, while areas with cooler colors (green-blue to cyan) have been experiencing decreasing temperatures. For precipitation, cooler colors represent increases and warmer colors represent decreases. Maps of the confidence trends are least-squares regression p-values, with purple areas representing statistically significant changes (p<0.05), yellow areas representing marginally significant changes (p<0.10), and grey areas representing changes that are not significant (p>0.10). Areas of white signify no data, or areas without sufficient climate station coverage to calculate climate change statistics.

We present climate trends calculated from the 0.5-degree resolution CRU TS 2.1 dataset [10] for 1951 to 2002 to identify those areas that have experienced the greatest rates of temperature and precipitation change (Figure 2). We chose this time period because high levels of anthropogenic greenhouse gasses were emitted during this 52-year period, it spans a long enough period for major environmental and ecological responses to climate change to have occurred, and the climate data are more robust for this period than earlier in the century. We recognize that the CRU TS 2.1 data were developed using methods that limit their suitability for trend analyses (see [11], and Discussion section “Use and misuse of climate data and analyses”), and the results should be interpreted in light of these limits. We also ran the Climate Wizard for a departure (anomaly) analysis that used future climate projections downscaled by Maurer et al. [12] to a 0.5-degree resolution for investigating how much temperature and precipitation is projected to change by 2070–2099 compared to a historical baseline in 1961–1990. A summary of the analysis results are presented below, but the complete results for many of these analyses can be further explored using the Climate Wizard interactive results web page located at: http://ClimateWizard.org (Figure S1).

### Hotspots for Recent Historical Climate Change Are in Central Asia, North Africa, and North America

Increases in average annual temperature at a rate of >0.2°C per decade during 1951–2002 occurred in northwestern North America, northern and western Africa, eastern Brazil, much of Europe, central Asia, and parts of southern and eastern Australia, as well as in other, more localized areas (Figure 2). Central Asia, northeastern Africa, and some pockets of northwestern North America have experienced temperature increases >0.4°C per decade—a rate of change that, if continued for a century, would greatly impact water, ecosystems, food, coasts and human health [1].

Mapping the p-values values for these regression trends shows that 58% of the Earth's terrestrial land area experienced statistically significant (p<0.05) annual mean temperature trends for the period 1951–2002, with 29% experiencing highly statistically significant change (p<0.001, Figure 2). Virtually all of these significant changes were temperature increases—only 0.2% of the area experienced significant temperature decreases.

**Figure 3:**
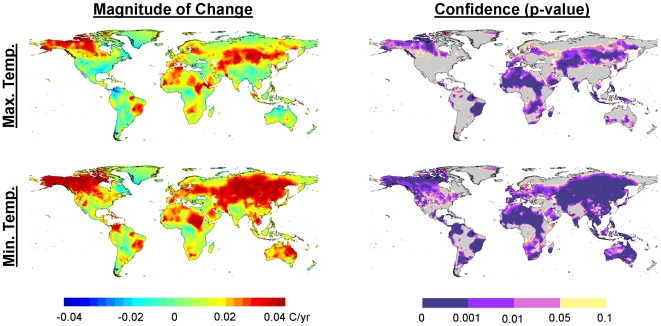
Minimum and maximum daily temperature change during 1951–2002. Maps of the magnitude (left) and confidence (right) for averaged minimum daily temperature (top) and maximum daily temperature (bottom) trends during 1951–2002 for each 0.5 degree grid cell. Warmer colors (yellow-green to red) represent increasing temperatures over time, while areas with cooler colors (green-blue to cyan) have been experiencing decreasing temperatures. Maps of the confidence trends are least-squares regression p-values, with purple areas representing statistically significant changes (p<0.05), and yellow areas representing marginally significant changes (p<0.10).

Average annual minimum daily temperature increases were greater than average annual maximum daily temperature increases. Minimum temperatures increased significantly over 69% of the Earth's terrestrial area, and decreased significantly over only 0.2% of the area, while maximum temperatures increased significantly over only 43% of the area, but decreased significantly over 1.0% of the area (Figure 3). In addition to a larger area experiencing increases in minimum temperatures, those minimum temperature increases were of greater magnitude than were the increases in maximum temperature. This differential in the diurnal temperature range (daily low temperature increasing faster than daily high temperature) has ramifications for snowpack [13]–[15], as well as crops that depend on winter chilling to synchronize pollination [16], [17].

Precipitation change between 1951 and 2002 also showed considerable spatial and temporal variation across the globe. Although globally, on average, precipitation showed no significant trend (p = 0.87), statistically significant (p<0.05) precipitation changes (both increases and decreases) occurred over 17.7% of the terrestrial area (Figure 2). Precipitation decreases occurred over 9.4% of this area, predominately in western Africa and the African Sahel, as well as northern India and eastern Alaska (Figure 2). Another 8.3% of the globe experienced significant precipitation increases, mostly located in northern Canada, south-central United States, Argentina, and north-western Australia. Depending on their location, timing and magnitude, changes in precipitation can affect the occurrence of droughts or floods—both of which can place ecosystems and humans at risk.

### Regional Patterns of Change

**Figure 4:**
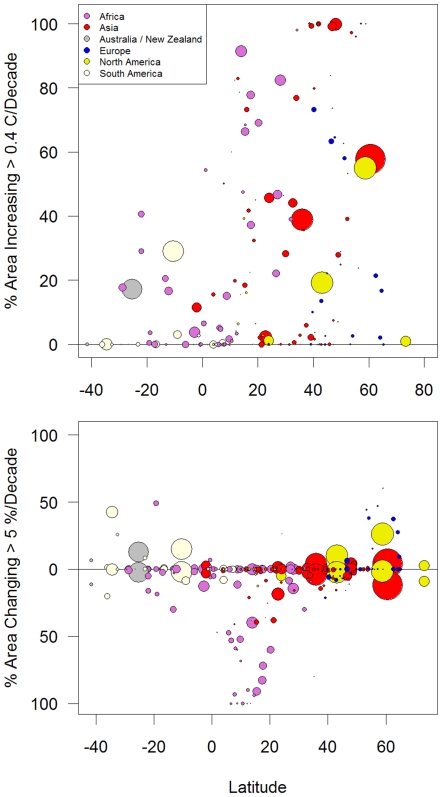
Temperature and precipitation change by country during 1951–2002. Percent area of countries experiencing annual mean temperature change >0.2°C/decade (top graph), and annual total precipitation change >5%/decade (above line) or <−5%/decade (below line, bottom graph) during 1951–2002 in the CRU TS 2.1 data set [10] plotted against the mean latitude of the country. Points are colored by continent and the size of the points is proportional to the land area of the country. See Figure S2 for a version of this graph with the countries labeled.

One of the strengths of the Climate Wizard tool is its ability to analyze and compare climate change among a set of many areas. Here we analyzed every country in the world (with the exception of some island countries) for mean temperature and precipitation change during 1951–2002. By plotting climate change as a function of the mean latitude of each country, we demonstrate that countries located in northern latitudes tended to have a greater proportion of their area (i.e., grid cells) experiencing significant annual mean temperature changes greater than 0.4°C/decade (Figure 4, top). For example, we found that all areas in the countries of Estonia, Latvia, Kazakhstan, Kyrgyzstan, Tajikistan, and Uzbekistan experienced temperature increases at a rate greater than 0.4°C/decade (Figure S2, top).

Precipitation changes also displayed a latitudinal pattern, with countries in latitudes between 0–20°N experiencing the greatest area of precipitation decrease at a rate greater than −5%/decade, while precipitation increases of this rate only occurred in countries either north or south of this latitudinal region (Figure 4, bottom). The specific countries with the greatest area experiencing significant precipitation decreases were predominately in West Africa, while the increases were predominately in northern Europe and South America (Figure S2b, bottom).

To further investigate these latitudinal trends, as well as to demonstrate the flexibility of the Climate Wizard tool, we analyzed major global latitudinal zones separately. Specifically, we analyzed changes in the Arctic Circle (above 66.57°N—at least one day of 24-hour sunlight or darkness), the northern temperate region (23.44°N to 66.57°N, between the Tropic of Cancer and the Arctic Circle), the tropical region (23.44°N to 23.44°S), and the southern temperate region (23.44°S to 66.56°S—between the Tropic of Capricorn and the Antarctic Circle). The highest rates of temperature increases have occurred in the northern temperate region (0.19°C/decade), and the lowest rates in the southern temperate region (0.11°C/decade) region.

One nuance found in these global climate patterns is that analyzing the area of significant change separately from the magnitude of change can provide different insights into how and where climate changes. For instance, when these latitudinal regions were analyzed with respect to the percentage of area having experienced significant change, we found that the southern temperate region experienced the greatest area of significant mean temperature change (62%). In contrast, only 36% of the arctic region experienced significant change, but the magnitude of overall change was greater in the arctic than in the southern temperate region (0.14°C/decade compared to 0.11°C/decade). This difference between the magnitude of change and area of significant change is a result of greater year-to-year variability in climate at more northern latitudes lowering the statistical significance while still maintaining a high overall magnitude of change. These results demonstrate that it is important to consider both the magnitude and statistical significance of a trend, because only relying on one or the other will not always be a good indicator of the environmental significance of the change being measured (see [18] and “Use and Misuse of Climate Data and Analyses” section for further discussion).

### Seasonal Patterns of Change

**Figure 5:**
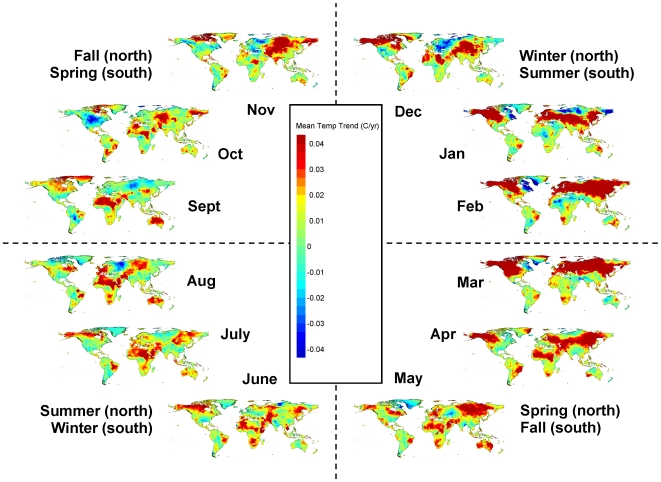
Monthly temperature changes during 1951–2002. Maps of the trend percent in mean temperature during 1951–2002 in the CRU TS 2.1 data set [10] for each month and broken up by season. These are least-squares regression coefficients calculated and mapped for each 0.5 degree grid cell. Areas with warmer colors (yellow-green to red) have been experiencing increasing temperatures, while areas with cooler colors (green-blue to cyan) have been experiencing decreasing temperatures.

**Figure 6:**
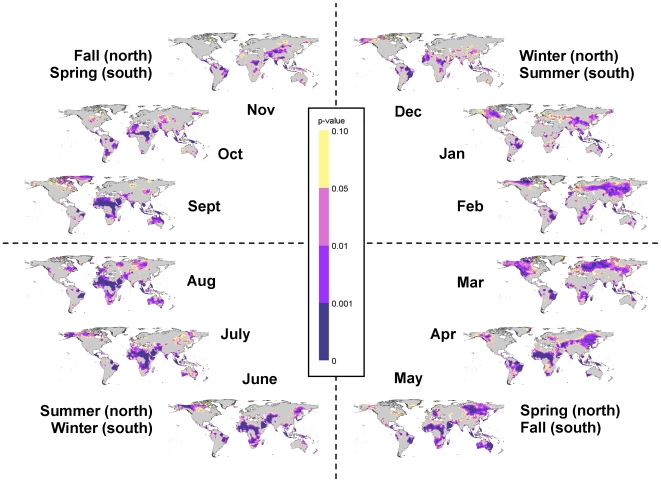
Confidence in monthly temperature changes during 1951–2002. Maps of the confidence in the mean temperature trends during 1951–2002 for each month (broken up by season). These are the restricted maximum likelihood p-values calculated and mapped for each 0.5 degree grid cell. Purple areas represent statistically significant changes (p<0.05), with the confidence in the darkest areas (p<0.001), yellow areas representing marginally significant changes (p<0.10), and grey areas representing changes that are not significant (p>0.10).

**Figure 7:**
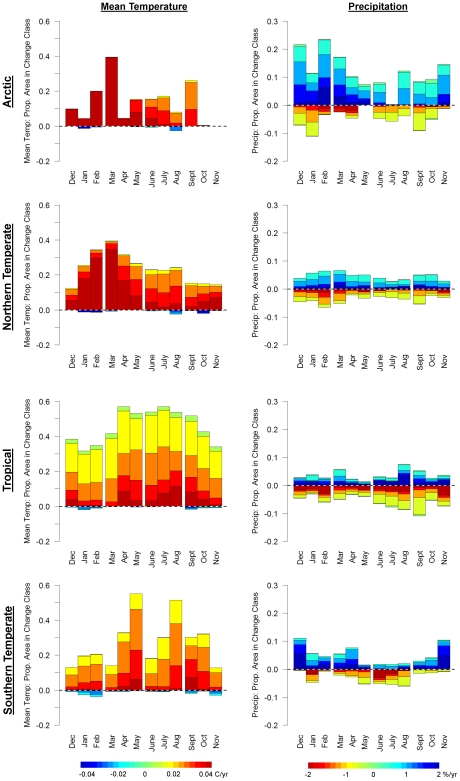
Monthly changes in temperature and precipitation by latitudinal climatic zones. The proportion of the area in (a) arctic, (c) northern temperate, (e) tropical, and (g) southern temperate climatic zones that have experienced significant (p<0.05) mean temperature and precipitation changes during 1951–2002 in the CRU TS 2.1 data set [10] colored by the magnitude of change from Figure 4. Increases are above the dashed line, decreases are below the dashed line.

Just as climate change varies spatially, it also varies seasonally. Targeted analyses of climate trends for specific months and seasons can be especially useful for evaluating the effects of climate change on specific ecological processes and ecosystem services. Using Climate Wizard, we analyzed 1951–2002 temperature trends globally, as well as within countries and latitudinal zones during each month (Figures 5 & 6) and during each of the four seasons (Figures S3). Analyzing monthly temperature trends within latitudinal zones indicates that the area of significant temperature change (of any magnitude) in the Northern Temperate zone was largest during January - April, peaking in March (Figure 7). In the Southern Temperate zone the largest area of significant temperature change tended to occur during July - October (Figure 7), which is the late winter and early spring in the southern hemisphere. However, the Tropical zone experienced greater area of change, but lower magnitude of change across months, as noted above (Figure 7).

**Figure 8:**
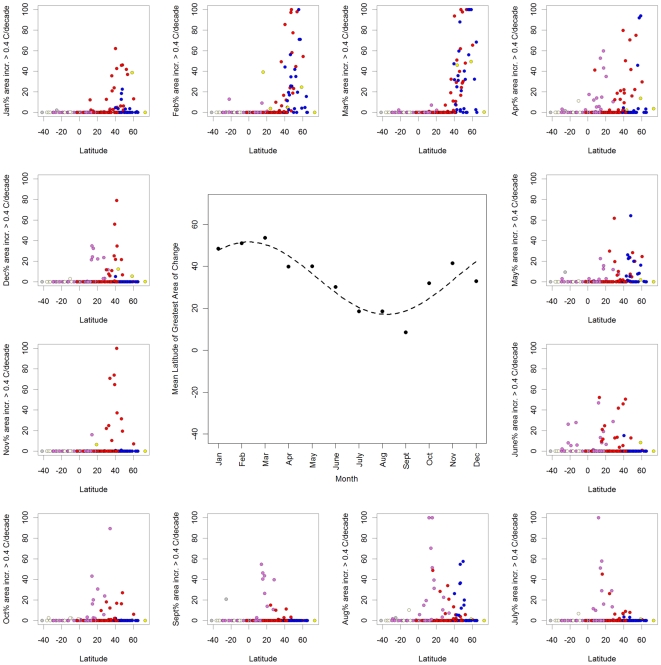
Latitudinal patterns of monthly temperature change by country. Percent area of countries experiencing mean temperature change >0.4°C/decade during 1951–2002 plotted against the mean latitude of the country for each month of the year, January–December (outside graphs starting from upper left). The center graph shows the mean latitude weighted by area of temperature increase >0.4°C/decade. A statistically significant (p<0.001) sinusoidal relation between month and the latitude with the greatest area of significant temperature increase >0.4°C/decade was found using nonlinear regression. Points are colored by continent (see Figure 2 for legend).

By analyzing monthly temperature changes within each country, the results similarly indicate that more northerly countries (between ∼30–60°N) tended to have the greatest area of significant change >0.4°C/decade during December - May (winter/spring in the northern hemisphere), while more southern and equatorial countries tended to experience the greatest temperature change during June - September (winter/spring in the southern hemisphere, Figure 8). Moreover, these Climate Wizard results allowed us to plot and identify specific countries that had the greatest proportion of their area with temperatures increasing at a rate of greater than 0.4°C/decade across all months, which indicated regional patterns such as countries in Asia tending to experience the greatest change from November - April, while countries in Europe experienced the greatest change during January - May (Figure 8). Summarizing this information revealed that the latitude with the greatest proportion of its area increasing at a rate >0.4°C/decade followed a sinusoidal pattern with respect to months ranging from 50°N during February-March to 10°N during August-September (Figure 8, p<0.001, nonlinear regression on country area weighted latitudinal means).

**Figure 9:**
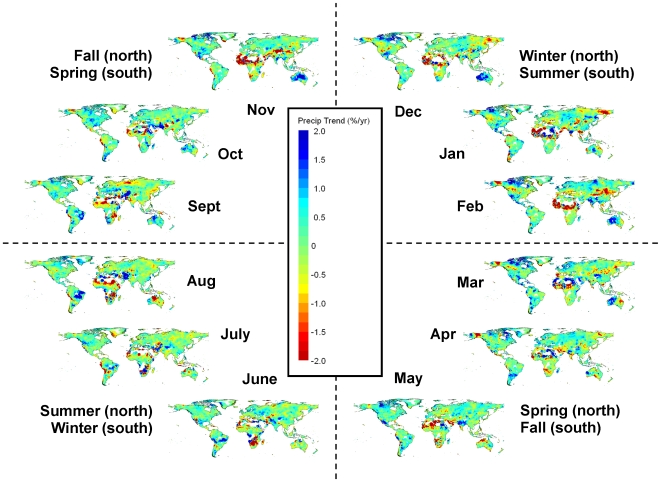
Monthly precipitation changes during 1951–2002. Maps of the trend in percent precipitation change during 1951–2002 for each month (broken up by season). These are least-squares regression coefficients calculated and mapped for each 0.5 degree grid cell. Areas with warmer colors (yellow-green to red) have been experiencing decreasing precipitation, while areas with cooler colors (green-blue to cyan) have been experiencing increasing precipitation.

**Figure 10:**
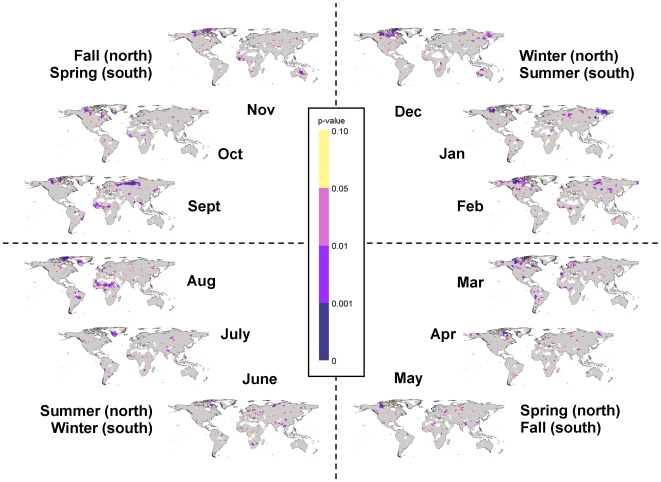
Confidence in monthly precipitation changes during 1951–2002. Maps of the confidence in the precipitation trends during 1951–2002 for each month (broken up by season). These are the restricted maximum likelihood p-values calculated and mapped for each 0.5 degree grid cell. Purple areas represent statistically significant changes (p<0.05), with the confidence in the darkest areas (p<0.001), yellow areas represent marginally significant changes (p<0.10), and gray areas indicate no significant change (P>0.1).

Precipitation exhibited some weaker seasonal patterns of change. For example, the Arctic zone experiencing the greatest precipitation increases during November–March, and the Tropical zone experienced the greatest precipitation decreases during July - September (Figures 7, 9 & 10, and Figure S4).

### Future Climate Projections

We used Climate Wizard for a departure (anomaly) analysis that compared modeled historic mean temperature and precipitation during 1961–1990 to future projected climate in 2070–2099 using downscaled projections from sixteen GCMs each run under three different greenhouse-gas emissions scenarios (48 projections [12]). The emissions scenarios analyzed include a global curbing of emissions over the next century (B1 scenario), a mid-21^st^ century leveling-off of emissions (A1B scenario), and a continual increasing rate of emissions over the 21^st^ century (A2 scenario; [19]). Because it is difficult to simultaneously interpret these 48 climate projections, we provide a summary of model ensemble results here, but the complete results for each projection can be explored at http://ClimateWizard.org.

**Figure 11:**
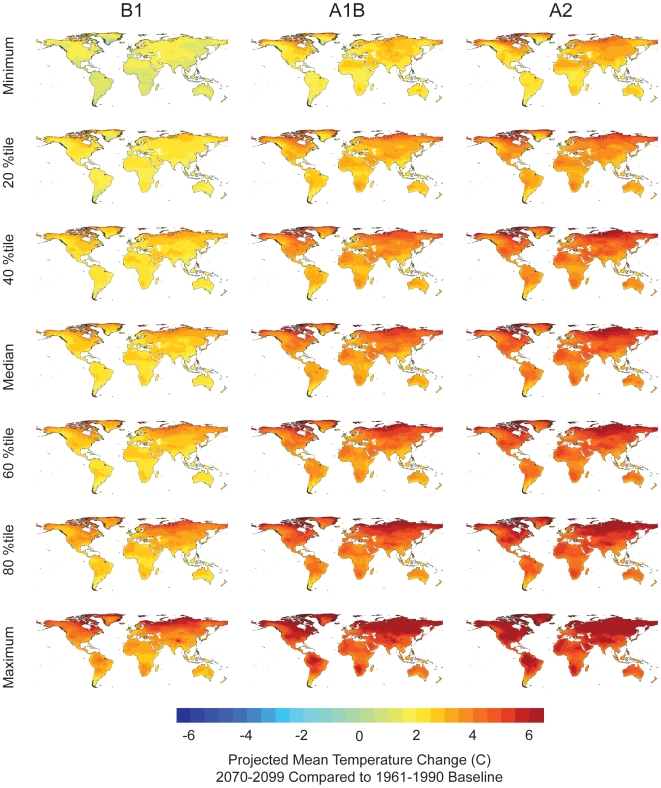
Projected temperature change ensemble analysis. Quantile ensemble analysis for mean temperature departures in 2070–2099 compared with 1961–1990. The three columns represent the three IPCC CO_2_ emissions scenarios (B1, A1B, A2), and the rows are the minimum, median, and maximum, as well as the 20, 40, 60, and 80^th^ percentiles projected by the 16 GCM models.

**Figure 12:**
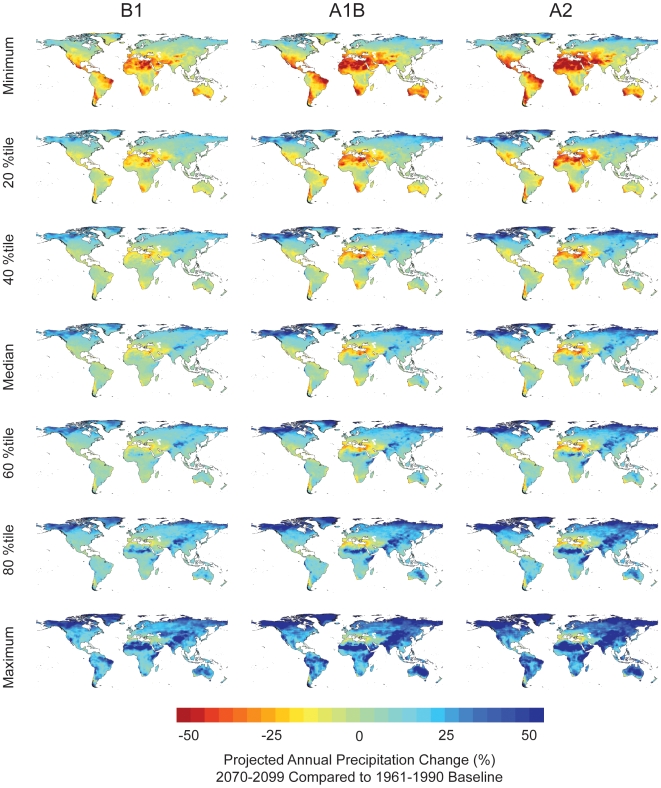
Projected precipitation change ensemble analysis. Quantile ensemble analysis for percent precipitation departures in 2070–2099 compared with 1961–1990. The three columns represent the three IPCC CO_2_ emissions scenarios (B1, A1B, A2), and the rows are the minimum, median, and maximum, as well as the 20, 40, 60, and 80^th^ percentiles projected by the 16 GCM models.

Climate Wizard uses a simple yet informative non-parametric (quantile-rank) model-ensemble approach that quantifies the range of future climate projections. This approach overlays all projections for a specific greenhouse-gas emissions scenario, then maps out specific quantiles/percentiles across the GCMs at each grid cell. Here we present the 0 (minimum), 20, 40, 50 (median), 60, 80, and 100^th^ (maximum) percentiles (Figures 11 & 12). While all models agree that mean temperatures will increase over all terrestrial land areas in the world (Figure 11), they often do not agree on the magnitude of that increase. Under the A2 scenario, specific grid cells in the 20^th^ percentile GCM ensemble ranged from 0.8–7.3°C, in the median GCM projection ranged from 1.7–8.4°C, and in the 80^th^ percentile GCM projection ranged from 1.9–9.5°C. Even though there is substantial variation between GCM projections across the globe, by examining the ensembles of models it becomes clear that the models tend to project that temperatures in areas in central North America, Northern Africa, Central Asia, and Western Australia will increase by the greatest amount (for a given emission scenario).

Precipitation projections are more complex to interpret than temperature projections because the GCMs often do not agree on whether precipitation will increase or decrease at specific locations, much less agree on the magnitude of that change. However, we can use the ensemble analysis to find model agreement at different percentile levels. For example, the median ensemble map can be used to identify areas where at least half the models project an increase or decrease in precipitation. In general, the majority of GCMs projected precipitation decreases in southern Europe, as well as parts of Africa, South America, southern Australia and southern North America and projected increases for most of the rest of the world. However, we can also identify areas where there is greater agreement between GCMs as to the direction of change. For example, under the A1B scenario, the Mediterranean region is projected to decrease in precipitation by approximately 10–30% in the 80^th^ percentile ensemble map (yellow-reddish colors), which means that 80% of the models (13 out of 16) agree that precipitation is projected to decrease by *at least* this amount (Figure 12). Moreover, some of the areas in this region are projected to decrease under the “Maximum” ensemble, showing that *all* GCMs project a decrease in precipitation these areas. Similarly, most of the arctic region is projected to increase in precipitation in the 20^th^ percentile ensemble map (bluish colors), showing that at least 80% of the GCMs agree that precipitation will increase there; parts of this region are projected to increase under the “Minimum” ensemble, showing that all models project an increase in precipitation in these areas (Figure 12). These two results of model agree for increasing precipitation and model agreement for decreasing precipitation can be overlaid to create a map identifying areas where at least 80% of the models agree precipitation will change in either a positive or negative direction (Figure S5).

**Figure 13:**
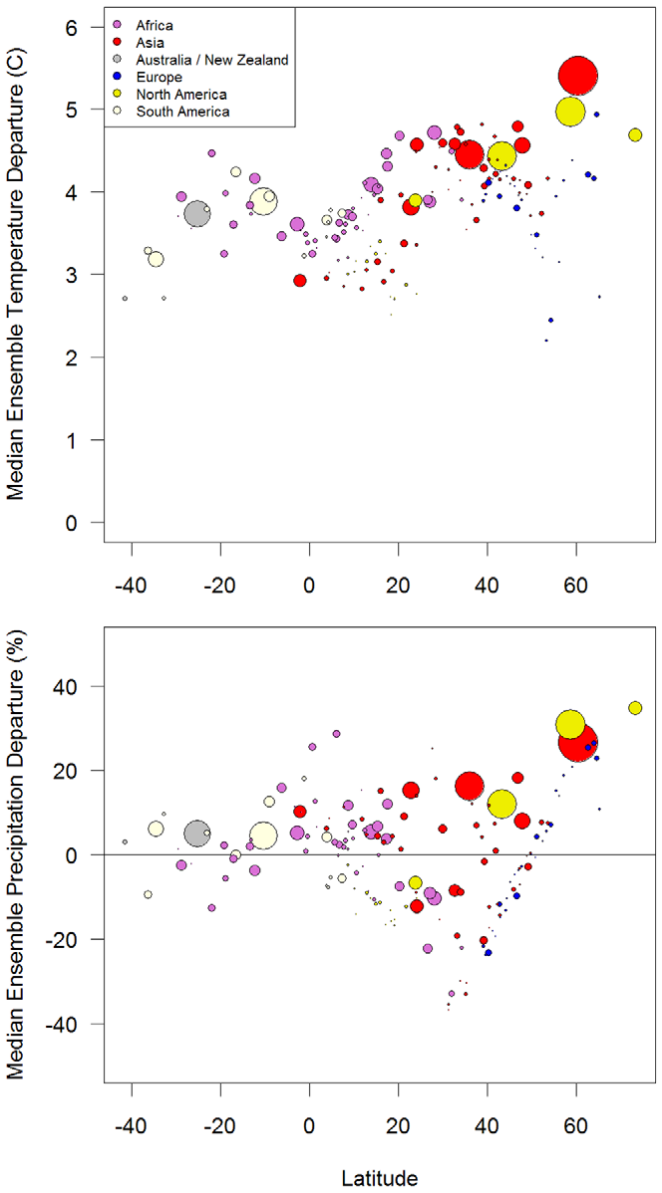
Projected temperature and precipitation change by country. Median ensemble projections (A2 emissions scenario) within each country in the world departures for temperature (top) and precipitation (bottom) in 2070–2099 compared with 1961–1990. Note that the model ensemble was computed at the country scale (not at the grid cell scale as was done in Figures 5 and 6), such that for each country only one GCM is used (the median GCM averaged over the entire country). Points are colored by continent and the size of the points is proportional to the land area of the country.

To further analyze these patterns, we used Climate Wizard to summarize the median ensemble projected mean temperature and percent precipitation departures within every country. This analysis shows regional and latitudinal patterns in these projected changes, such as the highest temperature increases projected at the higher latitudes in Asia and North America, but not in Europe (Figure 13, top). However, for precipitation, Europe showed a strong latitudinal pattern as indicated by the linear pattern of the blue points in Figure 13 (bottom) indicating projected increasing precipitation in northern Europe, and decreases in southern Europe.

**Figure 14:**
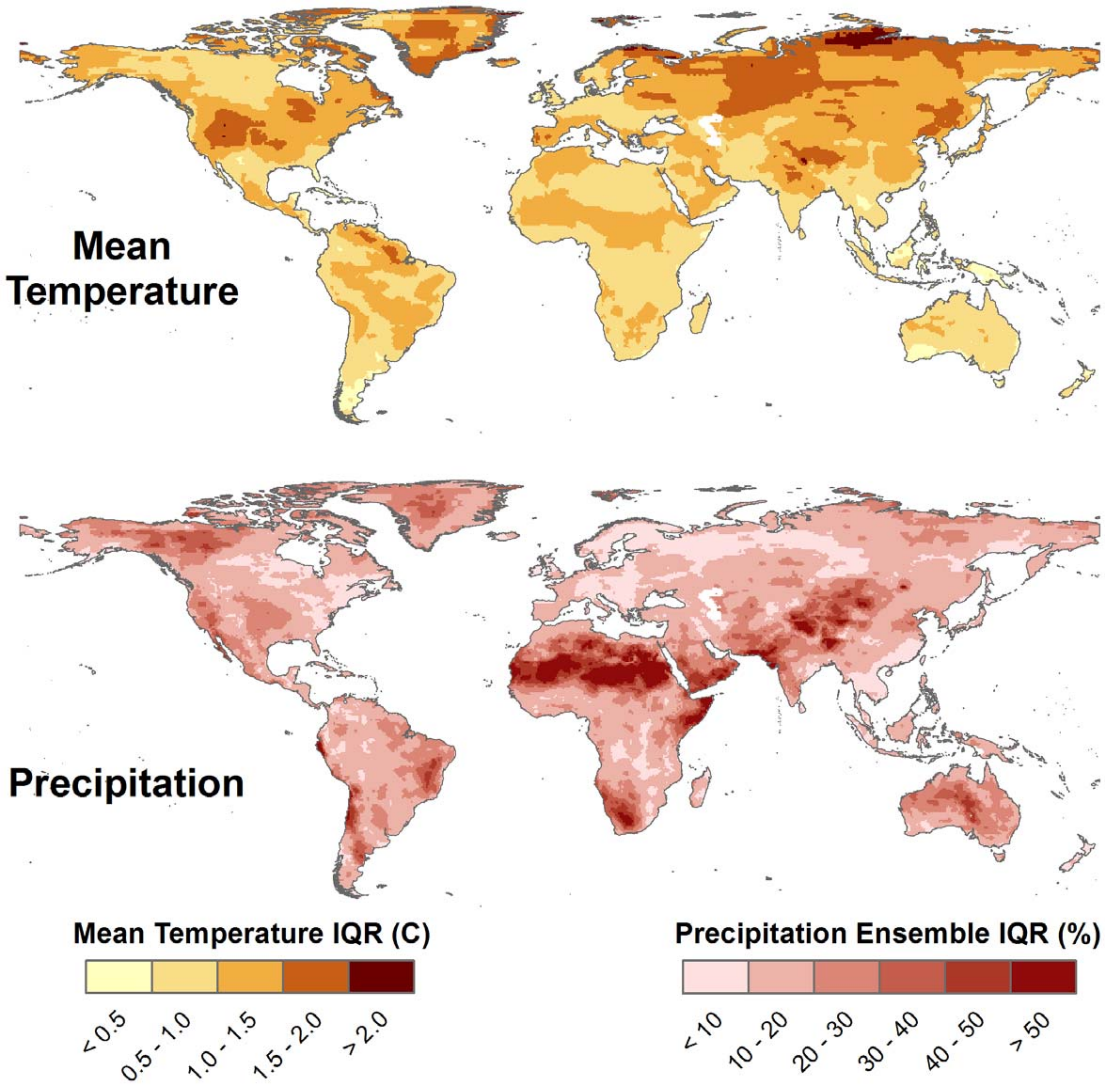
Variation in projected temperature and precipitation change between GCMs. Interquartile range of all 16 GCMs (A2 emissions scenario) at each grid cell for temperature (top) and precipitation (bottom) departures in 2070–2099 compared with 1961–1990. Lighter colors represent less variability between GCMs.

Finally, we used ensemble analysis to analyze the range of variability between GCMs by calculating the interquartile range (the difference between the 25^th^ and 75^th^ quantile maps). This non-parametric statistic is approximately equivalent to the parametric standard deviation, and can be used to show areas where there is greater or less variation between GCMs. Here we present this analysis for the A2 scenario (although other emissions scenarios produce similar patterns), which shows that the greatest between-GCM variation in temperature change projections occur in northern Asia, western Russia, northern and eastern Europe, Greenland, central North America, and northern South America (Figure 14, top). The patterns in GCM variation in percent precipitation change were quite different from those for mean temperature, with the greatest variation found in the Sahara, southern Africa, southern Middle East, and central Asia, among other areas of the globe (Figure 14, bottom).

## Discussion

This paper presents a framework and tool for practical climate-change analysis. The framework includes data sets (e.g. recent past climate and downscaled future GCM climate simulations), standardized methods (e.g. trend and departure analysis), and the tool produces useful products (e.g., maps, graphs and tables). We have demonstrated the analytic power of the Climate Wizard tool to analyze a set of different places (e.g., countries and latitudinal bands), both retrospectively and prospectively, and to summarize and visualize the results in innovative ways through maps, graphs, and an easy-to-use interactive mapping website (http://ClimateWizard.org). Our results are in general agreement with those reported elsewhere using similar data (e.g., [4]). Although other climate data analysis tools exist (e.g., the IPCC Data Distribution Centre Visualisation Tools, MAGICC/SCENGEN), the novelty and power of Climate Wizard is that it provides users with the flexibility to analyze any area of interest during any time period for which data are available (http://ClimateWizard.org/custom). Moreover, using internet technologies and web-based mapping, this tool provides climate-change visualization capabilities that are state-of-the-art.

### Using Climate Wizard to Identify Climate Impacts

The results presented here as well as other analysis results from the Climate Wizard can be used to identify past and projected future impacts due to climate change. The fact that much of the recent warming has occurred during late winter and early spring has important ecological implications. For example, the mean egg-laying date for tree swallows across North America is significantly negatively correlated with mean May temperatures [20]. It also appears that springtime warming in the western United States has led to increased fire activity [21], which can have many impacts, such as the over two thousand fires in California (USA) during 2008 that burned nearly 485,000 ha (1.2 million acres) and 511 structures, killed 15 people, and required over 25,000 firefighting personnel (http://www.fire.ca.gov). Because spring temperatures have increased more than the annual average temperature increases in both the northern and southern hemispheres, these ecological processes will be affected even more than would be expected from only analyzing annual climatic trends.

However, climate-change impacts to ecosystems and society often occur as a result of interactions between multiple climate variables. For example, by using the results of the analyses presented here to simultaneously consider both temperature and precipitation, we can gain greater insight into how potential future climatic changes may interact to alter ecological processes, such as soil moisture dynamics [22], fire occurrence [21], and carbon sequestration [23]. These results indicate that most of Africa, the Middle East, Asia, northeast South America, eastern Australia, and much of western North America became warmer and drier during 1951–2002. Using the flexibility of the Climate Wizard, this type of bivariate temperature-precipitation change analysis can be applied to specific months or seasons to target when during the year these changes are occurring.

### Extending the Climate Wizard Framework

The Climate Wizard framework is intended to be expanded and built upon, including different data sets, analysis techniques, and user interface applications. The Climate Wizard can analyze any gridded time-series of continuous data (i.e., not categorical) that is stored in the network Common Data Form (netCDF, http://www.unidata.ucar.edu/software/netcdf/). Because netCDF is the standardized way to store gridded time-series climate data, it is fairly straightforward to incorporate additional data sets into the Climate Wizard framework. These data sets could include simulations of global vegetation [24], fire [21], [25], water runoff [22], species range shifts [26], agriculture [27], sea level rise [28], heat stroke [29], disease [30], [31], and food security [32], [33]. Moreover, climate related “bioclimatic” variables could be calculated (e.g. rates of evapotranspiration, soil moisture availability, extreme climate statistics, plant hardiness measures, etc.), which act as metrics or surrogates for more specific climate-change mediated processes. In addition, climate-change analysis in marine systems could be incorporated into this framework and these same analytical techniques could be used to examine and explore how ocean temperatures, oxygen levels, and acidity have and are projected to change. At this point, the additional datasets must be manually added to the Climate Wizard, however, future research for the tool will focus on developing more streamlined and automated ways for seamlessly incorporating any gridded time-series netCDF dataset into the Climate Wizard framework.

Other tools can be created by using outputs from the Climate Wizard to produce user-friendly web “mash-up” applications that allow a user to dynamically interact with maps to visualize how climate changes. For example, programmers at Environmental Systems Research Institute (ESRI) are beginning to use the results of Climate Wizard analyses to create animated maps overlaid with The Nature Conservancy's (TNC's) conservation priority areas. This type of web application, for example, can allow users to zoom in on a specific conservation priority area, query to see how many endangered and imperiled species inhabit the area, see an animation of future projected temperature change at that location, and query points to see a graph of the projected temperature and precipitation change over time. Many more web mash-up applications could be created using technologies such as ESRI ArcGIS JavaScript API, ESRI ArcExplorer, Google Maps®/KML, Microsoft® Silverlight™, and Adobe® FLEX®.

### Use and Misuse of Climate Data and Analyses

The Climate Wizard provides easy access to a variety of climate data sets and analyses but, like any tool, it can be misused. We believe it is critical for Climate Wizard users to familiarize themselves with the strengths and limitations of the available data and the appropriateness of applying Climate Wizard's various analytical techniques to any particular data set. Climate Wizard website provides users with documentation and basic information about relevant publications, appropriate citations, and conditions-of-use restrictions for each data set. Here we provide an overview of important caveats in light of the analyses and results presented in this paper, although these caveats can be taken as more generally applicable to other data and analyses.

#### Climate data: observed data sets

Observed climate data have been used to create gridded time-series of climate data for many geographic areas [10], [34]–[36]. These estimated climate data are especially useful for practitioners interested in the climate of specific locations for which historical climate data are not available. A variety of gridded climate data sets have been created that range in spatial resolution, geographic extent, time period, and climate variables (e.g., [10], [37], [38]), and different data sets may contain different estimates of climate for the same location [39].

Moreover, the data sets in the Climate Wizard all have caveats that may limit their use for certain analyses and applications, and users are advised to carefully research a dataset before using it. For example, the CRU TS 2.1 global climate data (1901–2002) we used in our analyses were originally developed for use as input data for environmental models (http://www.cru.uea.ac.uk/~timm/grid/CRU_TS_2_1.html). For some regions and time periods, climate station data were insufficient for developing accurate climate estimates, particularly early in the twentieth century. To create complete spatial coverage, missing data for some grid cells were given the 1961–1990 mean climate values for certain time periods. This approach is described in the various publications accompanying the data (e.g., [10]) and the method produces a complete time series that can be used for many applications. However, in the Climate Wizard, we excluded years from the dataset that did not have at least one station within the threshold distance of 450 km (precipitation) or 1200 km (temperature) for each month [40], or did not have data within this threshold distance for 2/3 of the years being analyzed (see Methods and Materials section for more information). These data points were removed to decrease biases in the trend analyses. Other data sets have been developed for specific regions that use analytical methods to make gridded temperature and precipitation data sets temporally consistent (e.g., [38]). Daly [41] describes additional guidelines to use in determining whether an observed climate dataset is suitable for a particular application.

#### Statistical confidence in linear trends

We advise users of the Climate Wizard to interpret the linear trend climate change maps in relation to the respective map of statistical confidence (e.g., Figure 2). We recommend that areas with low statistical confidence in the rate of change (grey areas on map of statistical confidence) generally should not be used for making climate-related decisions. In addition, since historical climate maps are developed from weather station observations that have been spatially interpolated to create a seamless map of climate information, we recommend that single grid cells not be used for making climate-related decisions, but rather decisions should be based on many grid cells showing regional patterns of climate change with statistical confidence.

#### Climate data: downscaled future projections

It is important to remember that future climate simulations are projections of future climate, not accurate predictions of future climate change for any particular location or specific moment in time. There are many future climate projections produced by different GCMs and a number of different types of uncertainty that accompany these projections [42]. Randall et al. [43] describe some of the strengths and weaknesses of GCM simulations.

Because the spatial resolution of GCMs (2.5–3.5 degrees) is often too coarse for many scientific, management, and planning questions, a variety of methods have been developed for downscaling climate data to create finer spatial resolution data sets [44]. The downscaled global climate-change patterns we present in this paper at a 0.5 degree resolution (∼50-km) may still be too coarse for some applications, but regionally available, finer scale climate data sets can be incorporated into this framework. For example, we have recently added to the Climate Wizard future climate projections produced by 16 GCMs under three greenhouse-gas emissions scenarios, downscaled to 12-km resolution for the United States ([45]; see http://ClimateWizard.org). Increased spatial resolution, however, does not necessarily mean that data are more accurate. In some cases, the downscaling methods used to develop coarse-scale climate data are the same as those used to create fine-scale climate data, and these methods may ignore important processes that influence climate at regional and local scales [41].

Other aspects of the methods used to downscale climate data will influence the appropriate use and interpretation of the downscaled data. For example, the global 0.5 degree future projections used in Figures 11 and 12 were created by bias-correcting raw, coarse scale GCM output (following [7]) and spatially interpolating the bias-corrected monthly GCM departures to a 0.5 degree resolution grid, and then applying these interpolated data to a 0.5 degree grid of observed climate. This approach has a number of advantages, including that it incorporates the effects of topography on climate that are present in the 0.5 degree observed climate data. Statistical downscaling approaches assume stationarity in some aspects of the relationship of coarse-scale predictors to fine-scale climate. If future circulation patterns change in a way such that the past, observed relationship between coarse-scale and fine-scale climatic features is significantly changed, the resulting downscaled data would be less skillful at representing the future fine-scale climatic changes.

#### Using ensembles of multiple GCMs

The different GCMs often disagree in their projection of future climate. For temperature projections the GCMs generally only disagree in the magnitude of increase of temperature, however, for precipitation, the GCMs often disagree in both the direction of change (increasing vs. decreasing), as well as the magnitude. To best understand the range of future climate projections, we recommend using ensembles of multiple models to identify areas where models agree on climate change and where there is disagreement between models. Areas with severe disagreement between models should not be used for making climate-related decisions. A map showing areas where *at least* 80% of the GCMs agree precipitation will either increase or decrease (Figure S5) can be created easily created from Climate Wizard outputs by overlaying all positive values from the 20^th^ percentile precipitation map with all negative values from the 80^th^ percentile precipitation map (from Figure 12). See “Future climate projections” in the Results section for examples of using the Climate Wizard for ensemble analysis.

#### Spatial and temporal analyses

The Climate Wizard provides users with different analyses that can be applied to the available climate data sets. Some of the analyses, however, may not be appropriate for use with particular data sets, or for particular regions or time periods within a dataset. For example, as mentioned above in “Climate data: Observed data sets”, certain data sets may be less appropriate to use for time series analysis than other temporally consistent data sets [38].

Before performing analyses with the Climate Wizard, there are a number of issues that a user should consider. The time frame over which climate data are analyzed should be carefully selected to avoid drawing incorrect or biased conclusions that do not relate to the spatial and temporal scales at which the systems and processes of interest are operating [46]. The choice of the time period to analyze can influence the results of the analysis. For example, based on our analysis of the CRU TS 2.1 data, global mean temperature increased at 0.11°C/decade from 1941 to 2002, 0.16°C/decade from 1951 to 2002, 0.23°C/decade from 1961 to 2002, and 0.33°C/decade from 1971 to 2002 (Figure 1b). Thus, one's conclusions about the pace of warming could vary threefold depending on the time interval sampled. Trends also depend on location. Because climate varies spatially, the specific geographic region analyzed can greatly influence the results of a climate analysis. Some places are getting wetter while others are becoming drier. Some places have even experienced temperature decreases in the face of increasing global average temperatures.

#### Absolute change versus percent change

The Climate Wizard custom analysis tool can also be used to produce analyses of absolute precipitation change or percent precipitation change. Both types of analyses can be useful for looking at temporal and spatial patterns of climate change, but each require an understanding of the climate data used in the analysis. For example, the departure analyses for precipitation in Figure 12 are presented as the percent change between 2070–2099 and the 1961–1990 baseline period. Two grid cells may have the same simulated percent decrease in precipitation but a 10% decrease in precipitation for a grid cell in northern Africa that has relatively little annual precipitation may represent a much smaller absolute amount of precipitation change than a 10% decrease for a grid cell in the Brazilian Amazon region that receives a large amount of annual precipitation. Likewise, a grid cell in the African Sahel region, for example, may have the same percent change in precipitation during January and August, but because it rains much more during August due to the monsoonal rains, the absolute precipitation change would be much greater during August. Thus, the ecological effect of the same simulated future percent decrease in precipitation may be very different depending on a number of factors, including the region's total amount of annual precipitation, how the simulated precipitation decrease is distributed throughout the year, and the particular sensitivity to precipitation changes of the organism or system being studied. While we only presented percent change in precipitation, the Climate Wizard custom analysis tool has the ability to calculate either percent or absolute change in precipitation.

### Conclusion

Virtually all fields of study and parts of society—from ecological science and nature conservation, to global development, multinational corporations, and government bodies—need to know how climate change has and may impact specific locations of interest. Our ability to adapt to climate change depends on convenient tools that make past and projected climate trends available to planners, managers, and scientists at regional and local scales [2]. It is well known that climate has and will change differently across the globe, but it has been challenging for practitioners to analyze climate change within geographic areas relevant to specific scientific, management, or policy questions being addressed.

Many responses to climate change will require technological innovations. We believe Climate Wizard begins to meet this challenge in bridging a gap between the need for and accessibility of climate-change analyses by allowing a wide range of practitioners to explore how climate changes. Moreover, this tool can be adapted to develop web-based tools that are targeted at educating the general public about how climate change may affect specific human and natural systems—web-based applications as easy as Weather.com® to use and interpret. Polls of the American public consistently reveal that people do not have a sense of how climate change will affect their lives, and that they perceive climate change impacts as being more global and non-human, rather than affecting their families and local communities [47]. For this to change, information about past and future climate trends must be more widely and easily accessible to scientists and, moreover, the climate-change information scientists are producing must be communicated to planners, managers, and the general public. We have presented a step toward this end.

## Materials and Methods

The analyses in this paper were created using the Climate Wizard climate-change analysis toolbox, which is an integrated set of tools that access and analyze time-series climate surfaces (both past observed and future projected) at a range of spatial scales using a combination of geographic information systems (GIS), statistical analysis techniques, and web-based technologies (http://ClimateWizard.org). The core functions of the Climate Wizard tool are written in the statistical program R [9] that analyses climate data stored in netCDF file formats, but the tool uses the ArcGIS server software to identify the spatial area to analyze and to provide output data in GIS formats. A web mapping user-interface was developed to make the tool more accessible and to assist with both the input and output of data and information. The various components of the tool—climate data, change analysis, and user-interface—as well as how it was used to create the analyses presented in this paper are described below.

### Climate Data

The Climate Wizard does not create climate data—it uses climate data that have been produced by other researchers. The CRU TS 2.1 monthly climate dataset [10] was used to analyze recent historic climate change during 1951–2002. This dataset has a 0.5-degree spatial resolution (grid cells approximately 50 km per side, depending on latitude) and includes the following climate variables (summarized for each month): daily mean temperature (monthly average, °C), daily minimum temperature (monthly average, °C), daily maximum temperature (monthly average, °C), and precipitation (monthly total, mm). This dataset was developed based on historic records from thousands of climate stations around the world. The number of available climate station records in the CRU TS 2.1 dataset varies through time and, for certain time periods, some grid cells were not within the defined threshold distance of a station required to accurately estimate the temperature and/or precipitation [40] using the station data provided with the CRU TS 2.1 data [10]. For these time periods, the climate values in a grid cell were given the grid cell's 1961–1990 averages [10]. Therefore we excluded years from the dataset that did not have at least one station for each month within the threshold distance of 450 km (precipitation) or 1200 km (temperature) [40]. Then we excluded grid cells that did not have at least one station within the threshold distance for 2/3 of the years being analyzed. These data points were removed to decrease biases in the trend analyses. This ensured that any data that were given the 1961–1990 averages were excluded from the analysis, as well as ensured there were a sufficient number of years with data.

We used projections from 16 GCMs downscaled to a 0.5 degree resolution as in Maurer et al. [12] for monthly averages of daily mean temperature and monthly totals of precipitation for 1950–2099. This downscaling method uses the two-step bias correction spatial downscaling method, which has been applied extensively at regional, continental, and global scales [7], [45]. We used output from each GCM under three greenhouse gas emissions scenarios (A2, A1B, B1; [19]). All projections were generated for the World Climate Research Programme's (WCRP's) Coupled Model Intercomparison Project phase 3 (CMIP3) multi-model dataset [48] and used for analyses included in the IPCC Fourth Assessment Report [4]. While there have been investigations into weighting the output from some GCMs more heavily (e.g., [49]), recent findings suggest that including an ensemble of projections is more important than the weighting scheme [50], [51], thus we assume equal weighting of GCMs.

For both the past and future monthly climate data, we calculated annual and seasonal climate-change statistics for December-February (DJF), March-May (MAM), June-August (JJA), and September-November (SON). Annual and seasonal temperatures were calculated as the mean (weighted by the number of days per month and accounting for leap years) and annual and seasonal precipitation were calculated as the total amount (i.e., sum of the months). All base climate data were stored in netCDF file formats (http://www.unidata.ucar.edu/software/netcdf/) for use in Climate Wizard.

### Climate Change Analysis

#### Trend analysis

To estimate linear climate-change trends the Climate Wizard uses restricted maximum likelihood (REML) estimation assuming an AR1 time-series pattern in the residuals. This is computed using a generalized least squares method of the nlme contributed package to the R statistical software [9], [52]. The REML analysis was run for every grid cell. The data used in the trend calculations were accessed from the netCDF historical climate and GCM files using the ncdf contributed package to R [53]. The linear trends (*b*_o_) parameter values and p-values were calculated annually, seasonally, and for each climate variable, then mapped using the R contributed package maptree [54]. All trend rates are expressed as a change per decade (except where otherwise noted), and precipitation trends were calculated as the percent change from the average of the entire analysis period 1951–2002.

#### Departure/anomaly analysis

Departure analyses were calculated for all future projections. A departure analysis was calculated by first averaging the climate variable analyzed at each grid cell for the period from 1961–1990, creating a map of the baseline “normal” climate value at each grid cell. For each climate variable analyzed (mean temperature and precipitation), each of the 48 future climate projections were averaged at each grid cell for the time period 2070–2099, and the baseline value was subtracted from this average (at each grid cell). Precipitation change was calculated as the percent change from the baseline average, and temperature was calculated as the absolute change from the baseline average.

An ensemble analysis was conducted for each climate variable and emissions scenario combination. This was done for each emissions scenario by overlaying the 16 GCM departure maps and using the R statistical package command quantile to calculate the following ensemble quantile values *at each grid cell*: 0 (mathematical minimum projected change), 0.20, 0.40, 0.50 (median projected change), 0.6, 0.8, 1.0 (maximum projected change). These quantiles are equivalent to the 0, 20, 40, 50, 60, 80, and 100^th^ ensemble percentiles, respectively. The results of this analysis were mapped in R, producing maps of the ensemble minimum, median, and maximum change, as well as the 20, 40, 60, and 80^th^ percentiles, for each emissions scenario and climate variable combination. In addition, the 0.25 and 0.75 quantile ensembles were calculated and subtracted from each other resulting in maps of the ensemble inter-quartile range across the 16 GCMs (A2 scenario only) for both mean temperature and percent precipitation change. Then, to identify areas in the world where at least 80% of the GCMs agreed on the direction of precipitation change, we overlaid all positive values from the 20^th^ percentile ensemble precipitation change map with all negative values from the 80^th^ percentile ensemble precipitation change map.

#### Country-specific Climate Wizard analyses

All countries in the world were run through the Climate Wizard custom analysis to calculate historic linear trends in annual temperature and precipitation as well as monthly temperature trends, during 1951–2002. The average latitude of each country was calculated using GIS analysis. Graphs of country latitude versus proportion of the country with significant temperature increases >0.4°C/decade and significant precipitation changes were created using the statistical program R. For each month, the mean latitude of greatest area of temperature change >0.4°C/decade was identified by calculating average latitude of countries weighted by the area of significant temperature change >0.4°C/decade within each country. The latitude of greatest area of temperature change was plotted against month of the year and a nonlinear regression sine function was fitted to the data using the R statistical program contributed package nlme [52].

Similarly, all countries in the world were analyzed for future temperature and precipitation departures during 2070–2099 as compared to a baseline average of 1961–1990 for all 16 GCMs. An ensemble analyses was calculated that identified the median amount of change projected by the 16 models for each country. The latitude of each country was then plotted against the median projected change for both temperature and precipitation using the R statistical program.

### Computer Programming Notes and Web User Interface

The Climate Wizard tool is written as a combination of Python programming scripts (http://www.python.org), and R programming language scripts (http://www.r-project.org/). These scripts are linked together and served as a web service using ArcGIS Server (http://www.esri.com). The client to this web service is written in HTML/ASP.NET/JavaScript (http://www.w3.org/; http://www.asp.net/; http://en.wikipedia.org/wiki/JavaScript) web programming. The climate data are stored in the netCDF file format located on the remote computer server. This remote server is running the ArcGIS web-service linked to the HTML/ASP.NET/JavaScript, allowing users of this tool to access and analyze the data without having any program (except a web browser) or any climate data on their local machine. When a user uses the custom web page to requests an analysis, the grid cells to be analyzed are selected by an ArcGIS geoprocessesing analysis on the server, then these grid cells and the parameters for the climate-change analysis (e.g. climate variables, time period, time domain, type of analysis, etc.) are sent to R, where the analysis are run and graphics are created. Finally, a “results web page” is created using Python to generate HTML and JavaScript, which is customized to the analysis that was run. Once the analysis is complete (which may take minutes to hours depending on the complexity of the analysis), the user is e-mailed with a link to the results web page. From this results web page, the images of the maps and graphs, as well as the underlying GIS data sets can be downloaded by the user.

## Supporting Information

Figure S1 Climate Wizard interactive results web page.
(3.65 MB TIF)

Figure S2 Temperature and precipitation change by country during 1951–2002 (same as Figure 4, except countries with >20% area changing are labeled).
(6.00 MB TIF)

Figure S3 Seasonal temperature trends during 1951–2002. Both the magnitude of the trends (left) and p-value significance (right) of the trends are mapped out, and the area of significant change in each of the magnitude and p-value significance categories are provided at the bottom. The total height (positive plus negative) of the graph of trend magnitude is the area of significant (p<0.05) change, and the colors represent ranges of magnitude of change as represented in the maps above the graphs.
(2.07 MB TIF)

Figure S4 Seasonal precipitation trends during 1951–2002. Both the magnitude of the trends (left) and p-value significance (right) of the trends are mapped out, and the area of significant change in each of the magnitude and p-value significance categories are provided at the bottom. Terrestrial areas in white did not have sufficient station coverage for the trend analysis. The total height (positive plus negative) of the graph of trend magnitude is the area of significant (p<0.05) change, and the colors represent ranges of magnitude of change as represented in the maps above the graphs.
(2.10 MB TIF)

Figure S5 Model agreement in precipitation change. Map showing areas where at least 80% (13 of the 16 models) of the GCMs agree precipitation will either increase (blue areas) or decrease (brown areas). Areas in grey have less than 80% agreement in the direction of change in precipitation. Note that this map was created by overlaying all positive values from the 20th percentile precipitation map (from Figure 12) and all negative values from the 80th percentile precipitation map (from Figure 12).
(7.67 MB TIF)
