# Supplementary figures and images for: Applied Climate-Change Analysis: The Climate Wizard Tool
Source: PLoS One. 2009 Dec 15;4(12):e8320. doi: 10.1371/journal.pone.0008320 (PMC2790086; doi:10.1371/journal.pone.0008320)

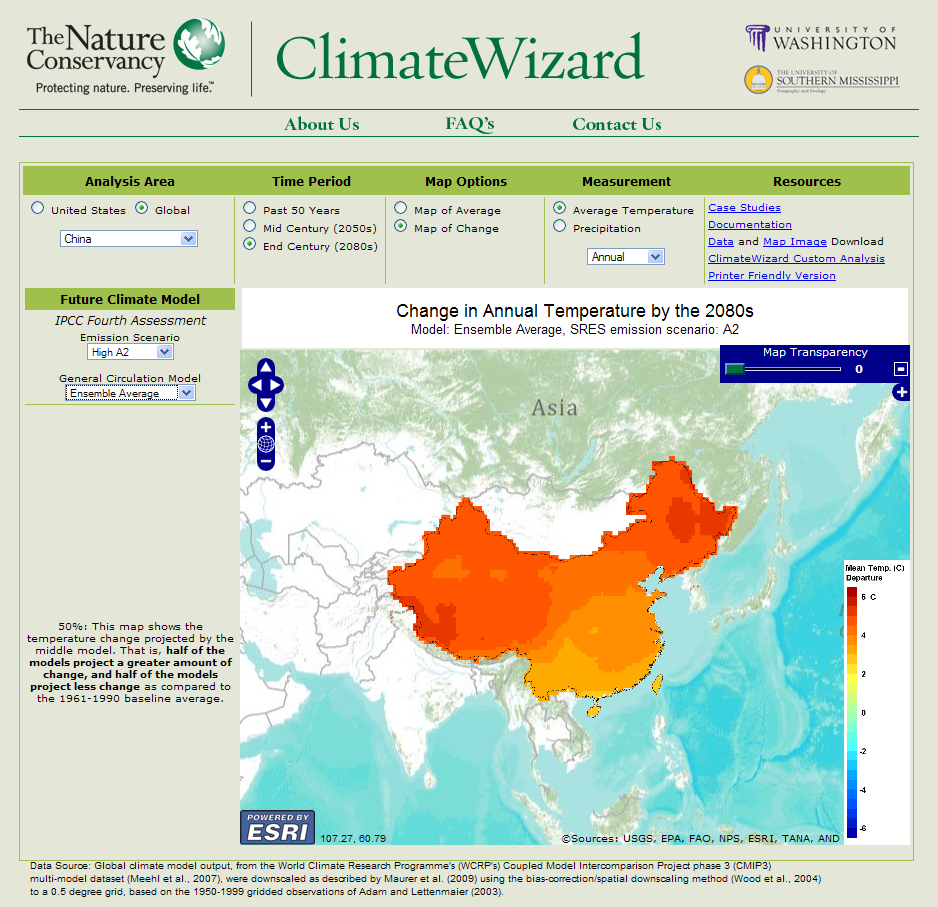

Supplement: Figure S1 — Climate Wizard interactive results web page. (3.65 MB TIF) [file pone.0008320.s001.tif]

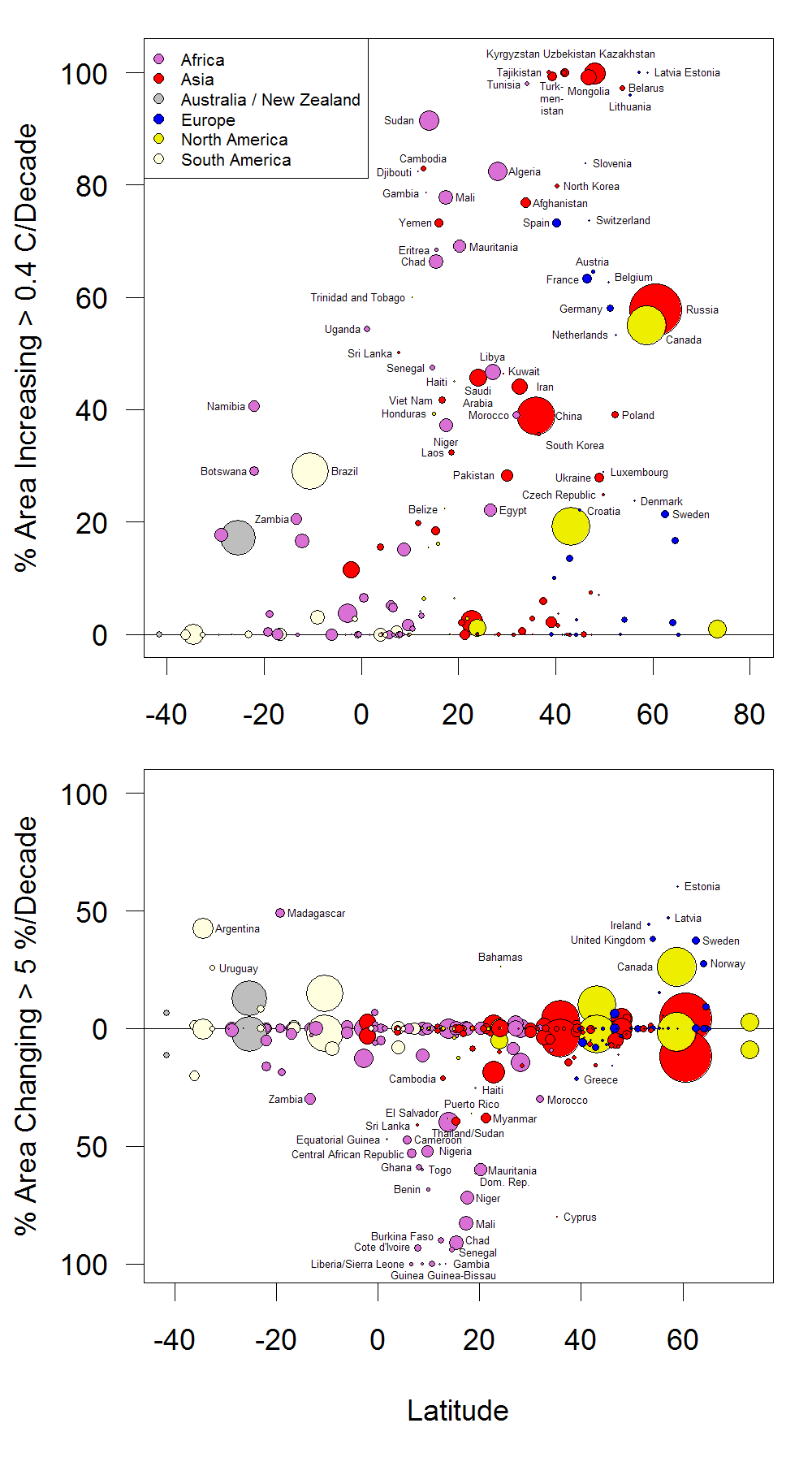

Supplement: Figure S2 — Temperature and precipitation change by country during 1951–2002 (same as Figure 4, except countries with >20% area changing are labeled). (6.00 MB TIF) [file pone.0008320.s002.tif]

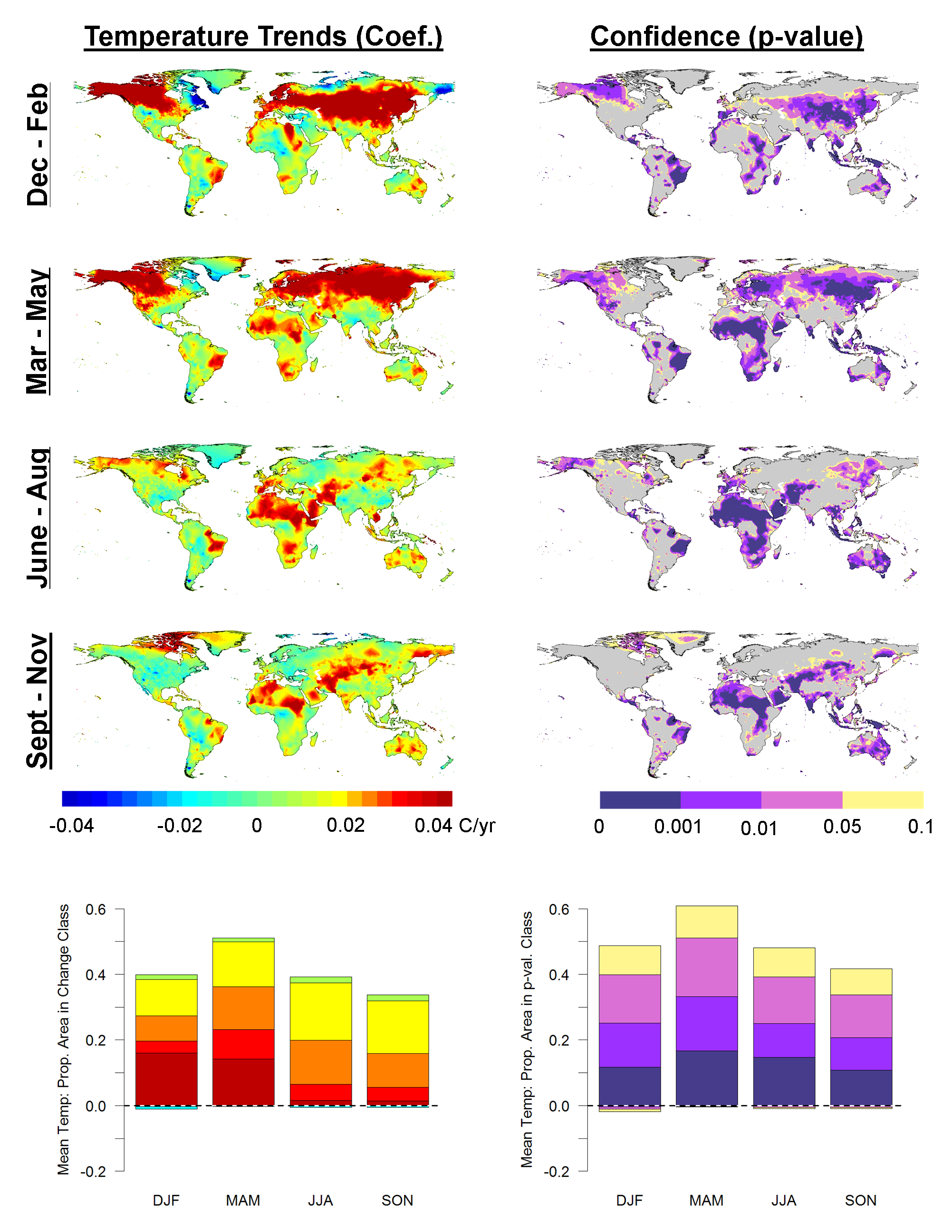

Supplement: Figure S3 — Seasonal temperature trends during 1951–2002. Both the magnitude of the trends (left) and p-value significance (right) of the trends are mapped out, and the area of significant change in each of the magnitude and p-value significance categories are provided at the bottom. The total height (positive plus negative) of the graph of trend magnitude is the area of significant (p<0.05) change, and the colors represent ranges of magnitude of change as represented in the maps above the graphs. (2.07 MB TIF) [file pone.0008320.s003.tif]

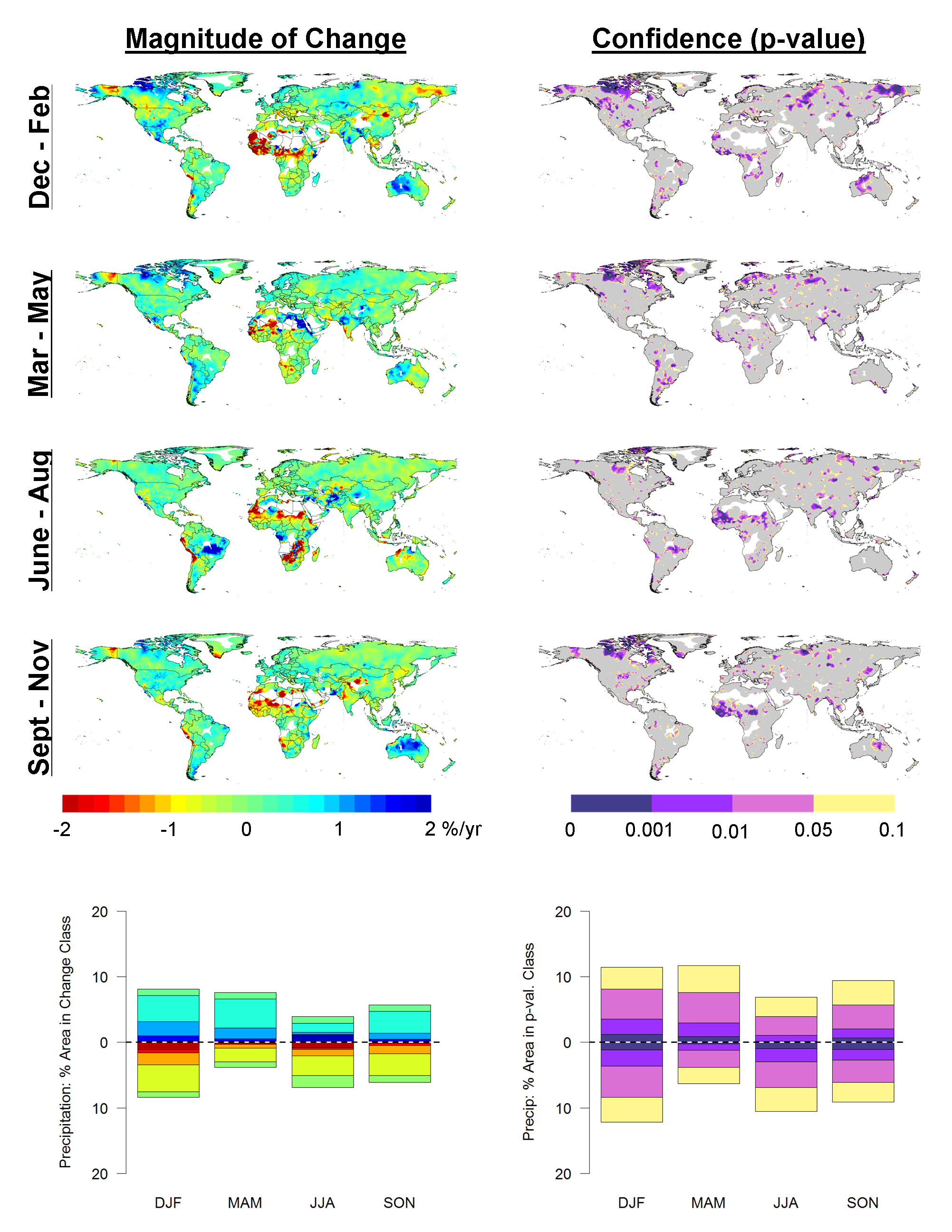

Supplement: Figure S4 — Seasonal precipitation trends during 1951–2002. Both the magnitude of the trends (left) and p-value significance (right) of the trends are mapped out, and the area of significant change in each of the magnitude and p-value significance categories are provided at the bottom. Terrestrial areas in white did not have sufficient station coverage for the trend analysis. The total height (positive plus negative) of the graph of trend magnitude is the area of significant (p<0.05) change, and the colors represent ranges of magnitude of change as represented in the maps above the graphs. (2.10 MB TIF) [file pone.0008320.s004.tif]

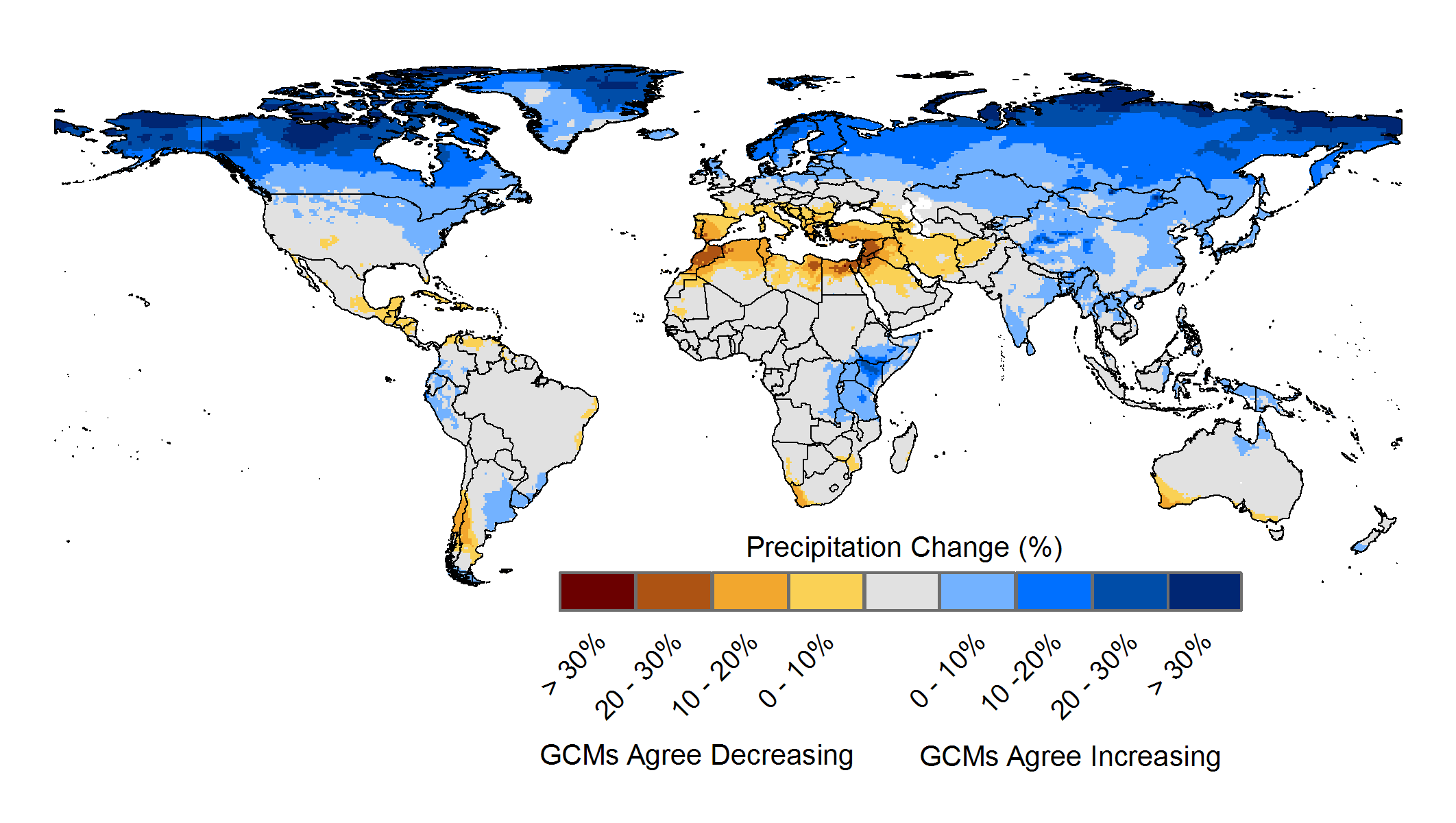

Supplement: Figure S5 — Model agreement in precipitation change. Map showing areas where at least 80% (13 of the 16 models) of the GCMs agree precipitation will either increase (blue areas) or decrease (brown areas). Areas in grey have less than 80% agreement in the direction of change in precipitation. Note that this map was created by overlaying all positive values from the 20th percentile precipitation map (from Figure 12) and all negative values from the 80th percentile precipitation map (from Figure 12). (7.67 MB TIF) [file pone.0008320.s005.tif]
